# 1030. Case Study. Optimizing Recovery: The Role of Occupational Therapy Across the Pediatric Burn Care Continuum

**DOI:** 10.1093/jbcr/irag033.546

**Published:** 2026-04-06

**Authors:** Amber Shojaie, Stephanie L Schneider

**Affiliations:** Children’s National Hospital, Washington, DC; Children’s National Hospital, Washington, DC

## Abstract

**Patient Presentation (age range, injury details, relevant history):**

This case of a pediatric patient with extensive trunk and axilla burns explores the significance of OT collaboration across all stages of care. OT interventions were initiated during the acute phase of injury in the intensive care unit, continuing through inpatient rehabilitation and outpatient follow-up, with an emphasis on communication among the therapists in all settings. Interventions included edema management, anti-contracture positioning, therapeutic exercises, scar management, participation in meaningful activities, community reintegration support, and caregiver training.

**Clinical Challenges:**

Patients with large total body surface area burns are at high risk for functional limitations due to pain, loss of range of motion (ROM), and scar development. The trunk and axilla in particular pose challenges given their impact on posture, breathing, upper extremity function, play, and activities of daily living (ADLs). Intensive and ongoing occupational therapy (OT) services across the continuum of care are essential for achieving long-term functional goals.

**Management Approach:**

Throughout the rehab continuum, the patient experienced both functional gains and temporary setbacks. With consistent OT interventions throughout recovery, the therapy team established a strong therapeutic alliance with the patient, which enhanced carryover of therapeutic goals. This continuity of care fostered increased engagement, leading to improved compliance of the therapy care plan. As a result, the patient demonstrated gains in ROM and independence in age-appropriate ADLs and play, contributing to successful reintegration into the home and community environments.

**Outcomes:**

This case highlights the importance of early and continuous OT, beginning in the acute phase and extending through outpatient care. Communication between clinicians across the continuum supported rapport, minimized contracture risk, optimized outcomes, and promoted meaningful participation for a pediatric patient with circumferential trunk and axilla burns.

**Lessons Learned:**

This case reinforces the vital role of skilled OT in pediatric burn care. It highlights the need for early anti-contracture positioning, caregiver training, progression of functional activities, and consistent communication between providers to support long-term recovery in children with large surface area burns.

**Applicability to Practice**:

